# Online scenario simulation teaching in airway management for undergraduate anesthesia students

**DOI:** 10.3389/fmed.2025.1563540

**Published:** 2025-06-10

**Authors:** Yun Lin, Ting-ting Wang, Yuan-yuan Hou, Xin-yu Lu, Le-jun Gao, Salad Abdirahman Hersi, Peng Gao, Qing-ping Wen

**Affiliations:** ^1^Department of Anesthesiology, First Affiliated Hospital of Dalian Medical University, Dalian, China; ^2^Department of Graduate, Dalian Medical University, Dalian, China; ^3^Department of Anesthesiology, Dalian Medical University, Dalian, China

**Keywords:** online teaching, scenario simulation teaching, anesthesia, airway management, undergraduate students

## Abstract

**Background:**

The rapid growth of online education has led to the extensive exploration of innovative teaching methods to improve learning outcomes in medical training. This study aimed to evaluate the effectiveness of online scenario-based simulation in an airway management course for undergraduate anesthesia students.

**Methods:**

A total of 130 undergraduate students participated in an online airway management course. The primary objective was to assess the effectiveness of this teaching method by comparing post-class quiz scores. Secondary outcomes were evaluated based on technical and non-technical skills scores across four simulation scenarios. An anonymous questionnaire was also distributed to gather students’ perceptions and experiences.

**Results:**

The simulation group exhibited a significant improvement in post-class quiz scores compared to the traditional group (*p* < 0.001). In the second simulation, students showed enhanced technical skills across all four scenarios (*p* = 0.030, *p* = 0.037, *p* = 0.028, *p* = 0.028, respectively), as well as improved non-technical skills, including task management, teamwork, communication, vigilance, crisis identification, decision-making, and self-confidence. Questionnaire responses indicated that students found the course both enjoyable and beneficial in improving their problem-solving abilities. Additionally, 97.3% of participants felt the course enhanced their self-learning and teamwork skills, while 97.22% reported it facilitated mastery of anesthesia techniques.

**Conclusion:**

Online scenario-based simulation teaching has proven to be a highly effective and engaging educational tool for undergraduate anesthesia students. It significantly improves both technical and non-technical skills while promoting critical thinking and problem-solving development.

## 1 Background

The rapid advancement of mobile Internet technology has positioned online medical education as a pivotal approach in medical training, primarily due to its flexibility and accessibility, which overcome the traditional limitations of time and space (1). This integration of medical education with online platforms has led to the emergence of various innovative educational models (2, 3). During the COVID-19 pandemic, our institution, like many others globally, shifted to online teaching (4). However, this transition presented significant challenges, especially in courses focusing on clinical skills. The conventional online teaching model often struggles to foster active student participation and engagement in the learning process (5, 6). Traditional teaching methods typically rely on one-way knowledge transmission through lectures, theoretical instruction, and limited video demonstrations, providing few opportunities for students to actively engage or participate in hands-on practice (7, 8). These challenges are amplified in an online setting, where the absence of immediate interaction and feedback—typical in face-to-face communication—further reduces student engagement (9). Given the highly practical nature of anesthesiology and the limited existing research on web-based anesthesia education, it is essential to explore and develop effective online teaching strategies for professional skills training to enhance the quality of teaching and learning in this domain.

Simulation training, which involves creating realistic medical scenarios where students assume various roles, is a valuable teaching method (10). It not only organizes and integrates theoretical knowledge but also effectively bridges the gap between foundational knowledge and clinical practice (11). Furthermore, it helps students enhance their teamwork, critical thinking, and clinical decision-making skills (12). Given the limited clinical experience of undergraduate students, which often results in challenges in applying knowledge flexibly and difficulties in crisis management and diagnosis (13), online simulation-based teaching can effectively address these gaps. Additionally, it can stimulate students’ interest in learning and improve their clinical reasoning and communication abilities (14, 15).

Students’ limited clinical experience was found to hinder their performance during initial simulation exercises in previous simulation-based teaching activities (13). To enhance the effectiveness of simulation training, a question-and-answer session was incorporated prior to the simulation activities. This session was designed to help students consolidate theoretical knowledge and prepare for the practical scenarios they would encounter. Additionally, debriefing is commonly integrated into the teaching process to optimize the outcomes of simulation training (16). In this phase, instructors guide students to critically evaluate the simulation process and review their clinical performance, fostering systematic and reflective learning while addressing areas for improvement (16, 17). This approach is particularly crucial in medical education, as it emphasizes collaboration, situational learning, and the development of presentation and decision-making skills (12, 18). With this method, students are more engaged in the online course, while instructors maintain greater control over the learning process, ultimately enhancing teaching quality (19).

Airway management is a critical responsibility for anesthesiologists. The identification and management of difficult airways present significant challenges, as inadequate management can lead to severe complications (20). To tackle these challenges, this study focused on teaching content related specifically to difficult airway management. Initially, students consolidated their theoretical knowledge of difficult airways through a question-and-answer session. Following this, online simulation teaching was implemented, incorporating effective debriefing techniques. In summary, this study aims to evaluate the efficacy of scenario-based simulation teaching in an online airway management course.

## 2 Materials and methods

### 2.1 Ethical approval and informed consent

The entire training process was conducted online via Tencent Meeting, with the simulation teaching facilitated through vital signs simulation software. No patients were involved or harmed during this study. The study was covered under the ethics review of the First Affiliated Hospital of Dalian Medical University and received approval from the teaching management of Dalian Medical University. The curriculum followed the teaching standards of Dalian Medical University. This study adhered to the Declaration of Helsinki and the ethical review guidelines of the First Affiliated Hospital of Dalian Medical University. All procedures were conducted in compliance with relevant guidelines and regulations, including but not limited to the use of online teaching platforms, confidentiality of student data, and privacy protection. Formal consent was obtained from all participants.

### 2.2 Sample and sample size

The sample size was calculated based on the primary outcome variable, measured by post-class quiz scores. According to our pilot study, the mean post-class quiz score in the traditional group was 29.03 with a standard deviation of 6.27. This study hypothesized a five-point difference in post-class quiz scores between the traditional group and the simulation group. Thus, a sample size of 84 students was required, assuming α = 0.05 and β = 0.05. To account for potential data loss, 130 participants were enrolled. All students were in their fourth year of university, with 65 participants in the simulation teaching group and 65 in the traditional teaching group. The sample size calculation was performed using PASS version 15.0.

### 2.3 Preparation phase

A total of 130 students participated in the study. All participants had previously completed the theoretical course in anesthesia and relevant training in tracheal intubation. Before the course, students were provided with study materials, including the latest airway management guidelines (20), in both Chinese and English. Each student was required to review these materials alongside the textbook before attending the class.

### 2.4 Pre-class quiz

**FIGURE 1:**
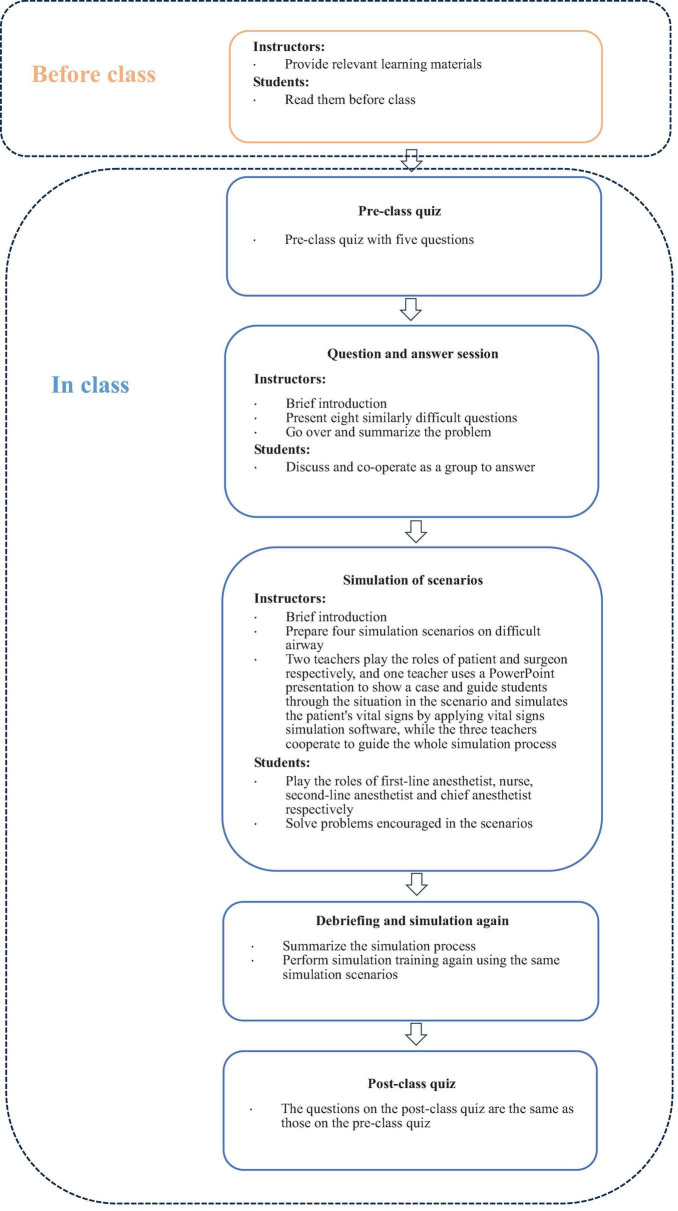
Implementation process of web-based anesthesia teaching study.

The detailed process of the web-based anesthesia teaching study is outlined in Figure 1. All students first completed a 20 min quiz assessing their knowledge of airway management. The quiz covered topics including: (1) how to perform airway assessments in patients with head and neck trauma; (2) preparation for tracheal intubation and extubation precautions in patients with pharyngeal tumors; (3) causes of hypoxia after anesthesia induction in patients with normal preoperative airway assessment; (4) measures to take after multiple failed intubations during general anesthesia induction. The instructor did not provide answers during the quiz, and the results were kept concealed. The same topics were retested at the end of the class to evaluate changes in students’ proficiency in professional knowledge.

### 2.5 Question and answer session

To enhance the effectiveness of simulation training, a question-and-answer session was introduced prior to the simulation exercises, aimed at reinforcing students’ theoretical knowledge and preparing them for the practical scenarios they would face. Students were randomly assigned to eight groups, each consisting of four members. Eight questions of comparable difficulty were prepared, with each group assigned one question to answer, and their responses were scored. Once a group completed its answer, other groups were invited to provide supplementary responses and earn points for correct answers. Following the session, detailed explanations were provided for each question. Topics covered included: (1) risk factors for difficult mask ventilation; (2) risk factors for difficult tracheal intubation; (3) methods for awake tracheal intubation; (4) strategies for managing anticipated difficult tracheal intubation; (5) grading of laryngoscopic exposure; (6) treatments for bronchospasm; (7) techniques for establishing an invasive airway; and (8) considerations for extubating patients with difficult tracheal intubation.

### 2.6 Scenario simulation training process

**FIGURE 2:**
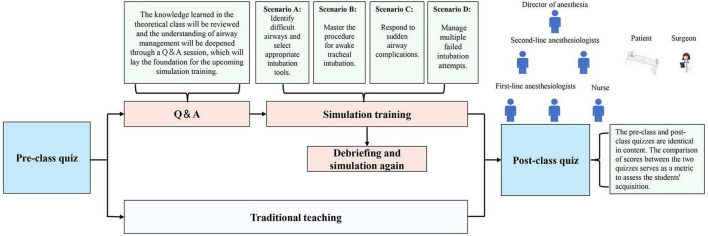
The structure and scenarios of the online simulation.

Prior to the training, roles were assigned to participants: two first-line anesthesiologists, two second-line anesthesiologists, an anesthesia director, and a nurse. To support the simulation, two instructors took on the roles of patient and surgeon, respectively (the structure and scenarios of the online simulation are illustrated in Figure 2). The instructor provided a thorough briefing on the simulation process, outlining the method and the responsibilities of each role. The simulation was designed to closely replicate the clinical workflow, including a preoperative patient visit and scenarios where first-line anesthesiologists sought assistance from second-line anesthesiologists or the director when encountering challenges. Four simulation scenarios were prepared: two for anticipated difficult airways and two for unanticipated difficult airways (Table 1). Virtual props, such as laryngoscopes, laryngeal masks, light sticks, fiberoptic bronchoscopes, and other relevant tools, as well as drugs used in simulations, were provided to the students. One instructor utilized a PowerPoint presentation to present case details (patient history, past medical history, relevant examinations, type of surgery, etc.) and a simulated training scenario in the operating room. Vital signs simulation software was employed to simulate the patient’s real-time vital signs, adjusting them based on the students’ actions. The other instructor played the role of the patient, simulating stress responses (e.g., choking, nausea, dyspnea) in reaction to the students’ interventions. The instructor guided the students through appropriate responses, utilizing PowerPoint to present emergency scenarios during the simulation (a demonstration of specific parts of the simulation is available in Supplementary Material 1). Students had the opportunity to communicate with both the patient and the surgeon at any point to gather information on the patient’s medical history and the surgical procedure. Each scenario lasted 20 min, followed by a teacher-led debriefing that addressed both technical and non-technical aspects of the simulation. Afterward, the same students participated in a second simulation. The instructor assessed and scored their performance across both simulations. Upon completion of each case, the instructor provided a comprehensive summary, including an analysis of any adverse patient effects and an evaluation of whether appropriate actions and precautions were taken.

**TABLE 1:** Simulation scenarios and technical and non-technical scoring criteria.

Scenarios	Technical skills	Non-technical skills
A) In the aftermath of a traumatic car accident, the patient sustained the cervical spine fracture combined with skull base fracture, then internal fixation will be per formed under general anesthesia. However, the patients limited head tilt and the mentum-to thyroid distance of less than 6cm pose challenges.	Identify difficult airway Fix cervical vertebrae and avoid nasotracheal intubation Select video-assisted laryngoscopy	Task management Teamwork Communication Sustained vigilance Crisis identification Decision making Self-confidence
B) In the case of a patient with ankylosing spondylitis undergoing laparoscopic gastrectomy under general anesthesia, the limited head tilt, the mentum-to thyroid distance of less than 6cm, and the mouth opening of less than 3cm all pose risks	Identify difficult airway Use awake tracheal intubation guided by fiber bronchoscopy Correct process of awake tracheal intubation	–
C) A radical thyroid surgery under general anesthesia is planned with no airway abnormalities on preoperative evaluation. Unfortunately, bronchospasm occurred due to multiple attempts at intubation after the failure of a direct laryngoscopic intubation	Attempt with the video-assisted laryngoscopy Seek assistance from a more experienced medical professional Solve bronchospasm	–
D) For a laparoscopic appendectomy under general anesthesia with no airway abnormalities on preoperative evaluation, direct laryngoscopic intubation failed and several alternative approaches were attempted without success.	Attempt with video-assisted laryngoscopy Seek assistance from a more experienced medical professional Find alternative approaches after three failed intubations (Laryngeal mask airway, wake up the patient, tracheotomy)	–

### 2.7 Evaluation of scenario simulation training

The instructor evaluated the entire simulation process using both technical and non-technical criteria. Technical points were awarded according to the following scoring system: two points for each completed exercise, two points for completing all exercises, one point for completing more than 50% of the exercises, and 0.5 points for completing less than 50%. Non-technical points were assessed based on the criteria established by the Stanford Anesthesia Cognitive Aid Group (21), covering aspects such as task management (e.g., task allocation, equipment preparation), teamwork, communication, sustained vigilance, crisis identification, decision-making, and self-confidence (non-technical criteria detailed in Supplementary Material 2). Each fully completed item earned 1 point, partially completed items were awarded 0.5 points, and incomplete items received 0 points. The detailed scoring scale for scenario simulation teaching is provided in Table 1.

### 2.8 Post-class quiz

A post-class quiz was administered at the end of the course, using the same questions as the pre-class quiz, to assess the effectiveness of the teaching modality by comparing pre- and post-class quiz scores.

### 2.9 Questionnaire

An anonymous online questionnaire was distributed to gather student feedback. The questionnaire included the following questions: (1) Are you satisfied with scenario simulation teaching? (2) How does this course compare to previous traditional courses? (3) Difficult airway simulation teaching feels incredibly real. (4) Does this course effectively improve problem-solving skills? (5) Does this course effectively improve self-learning ability? (6) Does this course effectively improve teamwork skills? (7) Does this course effectively increase interest in professional knowledge learning? (8) Difficult airway learning provides great benefits in improving anesthesia skills? (9) I am able to grasp the content of this course.

### 2.10 Statistical analysis

This study was a controlled trial, with students assigned to either the simulation or traditional teaching groups to evaluate the effectiveness of the online simulation. The primary objective was to assess the impact of the teaching modality by comparing post-class quiz scores. Secondary outcomes were evaluated based on technical and non-technical skills scores across two simulation scenarios. Data were presented as mean ± standard deviation. Post-class quiz scores were compared using a *t*-test. Both technical and non-technical skills in each of the four scenarios were analyzed using a paired *t*-test. Descriptive statistics were employed to summarize the questionnaire data, with frequency distributions and percentages used to illustrate the proportions within each category. A significance level of α = 0.05 was applied, with *p* < 0.05 considered statistically significant.

## 3 Results

### 3.1 Student basic information

Table 2 provides a comprehensive overview of participant information. All students had successfully completed theoretical coursework and practical exams; however, they had not undergone clinical practice.

**TABLE 2:** Demographic characteristics of participants.

Variable	Number	Percentage (%)
**Gender**		
Female	86	66.2
Male	44	33.8
**Age**		
25	2	1.5
24	6	4.6
23	22	16.9
22	73	56.2
21	27	20.7
**Normal training phase**		
Theory qualified	130	100
Technical qualified	130	100
**Clinical experiences**		
Yes	0	0
No	130	100

### 3.2 Web-based anesthesia simulation teaching enhanced students’ understanding of airway management

**FIGURE 3:**
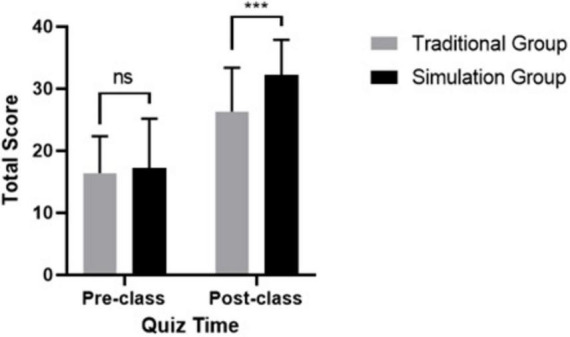
Comparison of post-class quiz scores between traditional group and simulation group. Post-class quiz scores are presented as means ± SD. Significantly different from the simulation group at ***P < 0.001.

Figure 3 illustrates the quiz scores of 130 students, showing a significant increase in post-class scores for the simulation group compared to the traditional group (*p* < 0.001). There were no significant differences in pre-class scores.

### 3.3 Web-based anesthesia simulation teaching demonstrated high effectiveness

Table 3 presents the evaluation of technical and non-technical points across four scenarios: two anticipated difficult airway scenarios and two unanticipated difficult airway scenarios. While no significant differences were observed in technical and non-technical points across the scenarios, the total scores (technical + non-technical) for all four scenarios demonstrated a statistically significant improvement during the second attempt compared to the first (*p* < 0.05).

**TABLE 3:** Comparing performance in the first and second simulations.

Classification	Term	First time	Second time	*P*-value
Scenario A	Technical points	2.25 ± 0.354	5.00 ± 0.000	0.058
	Non-technical points	2.25 ± 0.354	4.75 ± 1.061	0.126
	Overall score	4.50 ± 0.707	9.75 ± 1.061	0.030
Scenario B	Technical points	2.75 ± 1.061	4.50 ± 0.707	0.090
	Non-technical points	3.00 ± 0.000	5.50 ± 0.707	0.126
	Overall score	5.75 ± 1.061	10.00 ±1.414	0.037
Scenario C	Technical points	3.00 ± 0.000	5.75 ± 0.354	0.058
	Non-technical points	1.75 ± 0.354	4.75 ± 0.354	0.105
	Overall score	4.75 ± 0.354	10.50 ± 0.000	0.028
Scenario D	Technical points	2.75 ± 1.061	5.25 ± 0.354	0.126
	Non-technical points	2.25 ± 0.354	5.50 ± 0.707	0.144
	Overall score	5.00 ± 0.707	10.75 ± 1.061	0.028

### 3.4 Course feedback and satisfaction survey results

**FIGURE 4:**
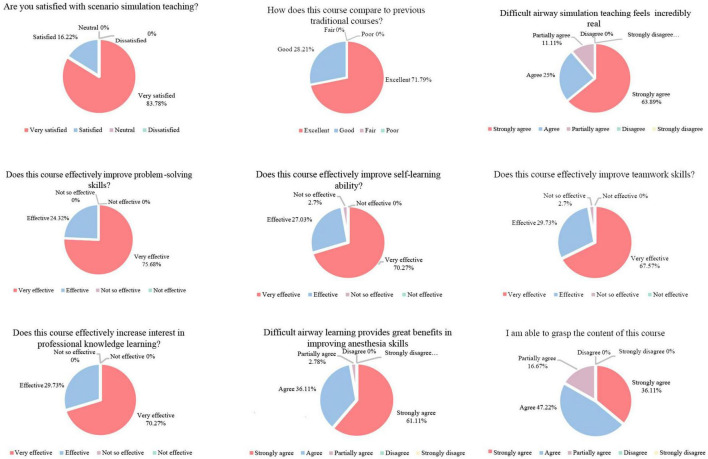
Questionnaire results on course feedback.

The survey results (Figure 4) revealed that all students expressed a preference for scenario simulation teaching, finding it superior and more engaging than their previous learning methods. The students highly valued this teaching approach. Specifically, 88.89% of participants found the simulation scenarios in the airway management course to be realistic. Additionally, 97.3% of students reported that the course enhanced their self-learning and teamwork skills, while 97.22% indicated that it improved their abilities in difficult airway management. A majority of students (83.33%) felt confident in their mastery of the course content. Overall, the survey responses unanimously indicated that the course effectively fostered independent exploration and problem-solving skills, with students subjectively reporting that this teaching method positively impacted their learning outcomes.

## 4 Discussion

The COVID-19 pandemic has profoundly reshaped the landscape of medical education, accelerating the widespread adoption of online teaching methods (22). While traditional online approaches offer increased accessibility, they often struggle to engage students effectively and fail to foster clinical reasoning (23), particularly in hands-on fields like anesthesia. Online scenario simulation teaching presents a promising solution to these limitations (23).

Simulation-based learning, long valued for its ability to replicate realistic clinical scenarios, allows students to apply theoretical knowledge in a controlled, risk-free environment (12). By integrating online platforms with simulation training, the flexibility and accessibility of the Internet can be combined with the interactive and immersive nature of simulation exercises (24). This hybrid approach not only enhances students’ technical competencies but also promotes the development of essential non-technical skills, such as teamwork, communication, and crisis management (12, 18, 25). Online simulation training offers students the opportunity to encounter and manage a variety of airway scenarios, including both anticipated and unanticipated difficult airways, without the risks associated with real clinical settings (26). For undergraduate students preparing to enter clinical practice, this simulation course equips them with the confidence needed to navigate complex clinical situations in their future careers (27). Moreover, the online format eliminates the need for physical presence in a simulation center, increasing accessibility for a larger number of students while reducing costs and enhancing convenience compared to traditional simulation training (24).

To maximize the effectiveness of simulation training, we facilitated students’ consolidation of knowledge related to difficult airways by posing a series of open-ended questions prior to the simulation exercises. This method not only fostered critical thinking but also maintained high levels of student engagement and motivation throughout the training (28, 29).

Our findings indicated that web-based anesthesia simulations significantly enhanced students’ understanding of airway management, facilitated the translation of theoretical knowledge into practical skills, and improved performance in subsequent simulations. Additionally, non-technical skills—such as task management, teamwork, communication, sustained vigilance, crisis identification, decision-making, and self-confidence—also showed marked improvement. The benefits of this simulation training included increased confidence, reduced anxiety, and improved proficiency (30). In the present study, post-class quiz scores from the simulation group significantly outperformed those from the traditional group, confirming the effectiveness of scenario simulation training.

Questionnaire results revealed that all students found the approach beneficial, enhancing their learning efficiency and interest in systematic professional development. Specifically, 97.3% of students reported improvements in self-learning and teamwork skills, while 97.22% felt the course enhanced their difficult airway management capabilities and believed the teaching method positively impacted their learning outcomes. Through the integration of online teaching and scenario simulation, undergraduate anesthesia students developed essential crisis management skills, crucial for addressing practical clinical challenges.

Overall, our findings indicate that online scenario simulation training is both highly effective and engaging for anesthesia education. These results align with studies in nursing and pharmacy education, which have shown that online simulation teaching improves students’ learning outcomes and offers a positive educational experience (31, 32). Furthermore, students have demonstrated enhanced execution and leadership skills in crisis situations (33). Another study found that students involved in online simulation-based teaching exhibited significant improvements in knowledge acquisition, with the interactive nature of simulation-based learning proving particularly effective in fostering enthusiasm for the subject matter (34).

In online simulation teaching, active student engagement is essential. However, instructors often face challenges in closely monitoring student involvement and accurately assessing participation rates in an online environment (35). This lack of control can directly affect the quality of instruction and hinder the achievement of desired learning outcomes (18). Direct debriefing effectively addresses these challenges by extending teaching sessions, motivating students, and enhancing instructors’ control over the online classroom (36). Based on the learning outcomes observed during the direct debriefing phase, instructors can guide students back into simulation scenarios, help them identify areas of weakness, supplement their knowledge, and integrate it into a coherent framework (37). Additionally, debriefing encourages students to articulate their thoughts and emotions, promoting reflection on both individual and collective experiences (38).

Despite these promising results, several limitations exist in this study. First, the absence of blinding led to potential bias in the analysis of subjective observations. Second, the study did not include follow-up assessments to evaluate the long-term impact of online simulation training on students’ clinical practice. Future work will involve tracking these students’ progress over the long term to assess the continued influence of this teaching method. Additionally, multicenter, randomized controlled trials with larger sample sizes will be conducted to further investigate the effectiveness of this approach.

## 5 Conclusion

In conclusion, online scenario simulation training has proven to be a highly effective and engaging educational tool for undergraduate anesthesia students. Incorporating this training into the curriculum not only enhances students’ technical and non-technical skills but also fosters the development of critical thinking and problem-solving abilities.

## Data Availability

The datasets analyzed in this study are available from the corresponding authors on a reasonable request.

## References

[1] Chandran V Balakrishnan A Rashid M Pai Kulyadi G Khan S Devi E Mobile applications in medical education: A systematic review and meta-analysis. *PLoS One.* (2022) 17:e0265927. 10.1371/journal.pone.0265927 35324994 PMC8947018

[2] Almutawa J Nicolaou C. Medical students’ view on enhancing engagement in online teaching. *Med Teach.* (2022) 44:697. 10.1080/0142159X.2021.1970733 34459331

[3] Chang M Liao M Lue J Yeh C. The impact of asynchronous online anatomy teaching and smaller learning groups in the anatomy laboratory on medical students’ performance during the Covid-19 pandemic. *Anat Sci Educ.* (2022) 15:476–92. 10.1002/ase.2179 35291048 PMC9082485

[4] Chan E Khong M Torda A Tanner J Velan G Wong G. Medical teachers’ experience of emergency remote teaching during the COVID-19 pandemic: A cross-institutional study. *BMC Med Educ.* (2022) 22:303. 10.1186/s12909-022-03367-x 35449047 PMC9021818

[5] Kaup S Jain R Shivalli S Pandey S Kaup S. Sustaining academics during COVID-19 pandemic: The role of online teaching-learning. *Indian J Ophthalmol.* (2020) 68:1220–1. 10.4103/ijo.IJO_1241_20 32461490 PMC7508127

[6] Longhurst G Stone D Dulohery K Scully D Campbell T Smith C. Strength, weakness, opportunity, threat (SWOT) analysis of the adaptations to anatomical education in the United Kingdom and republic of ireland in response to the covid-19 Pandemic. *Anat Sci Educ.* (2020) 13:301–11. 10.1002/ase.1967 32306550 PMC7264742

[7] Michael J. Where’s the evidence that active learning works? *Adv Physiol Educ.* (2006) 30:159–67. 10.1152/advan.00053.2006 17108243

[8] Rossi I de Lima J Sabatke B Nunes M Ramirez G Ramirez M. Active learning tools improve the learning outcomes, scientific attitude, and critical thinking in higher education: Experiences in an online course during the COVID-19 pandemic. *Biochem Mol Biol Educ.* (2021) 49:888–903. 10.1002/bmb.21574 34652877 PMC8653153

[9] Wilcha R. Effectiveness of virtual medical teaching during the COVID-19 crisis: Systematic review. *JMIR Med Educ.* (2020) 6:e20963. 10.2196/20963 33106227 PMC7682786

[10] Gong T Wang Y Pu H Yin L Zhou M. Study on the application value of PBL combined with situational simulation teaching method in clinical practice teaching of radiology department. *Comput Math Methods Med.* (2022) 2022:6808648. 10.1155/2022/6808648 35991150 PMC9388287

[11] Koukourikos K Tsaloglidou A Kourkouta L Papathanasiou I Iliadis C Fratzana A Simulation in clinical nursing education. *Acta Inform Med.* (2021) 29:15–20. 10.5455/aim.2021.29.15-20 34012208 PMC8116070

[12] Seam N Lee A Vennero M Emlet L. Simulation training in the ICU. *Chest.* (2019) 156:1223–33. 10.1016/j.chest.2019.07.011 31374210 PMC6945651

[13] Gao P Wang C Liu S Tran K Wen Q. Simulation of operating room crisis management - hypotension training for pre-clinical students. *BMC Med Educ.* (2021) 21:60. 10.1186/s12909-020-02477-8 33461550 PMC7814582

[14] Hofmann H Harding C Youm J Wiechmann W. Virtual bedside teaching rounds with patients with COVID-19. *Med Educ.* (2020) 54:959–60. 10.1111/medu.14223 32403185 PMC7273015

[15] Chandra S Laoteppitaks C Mingioni N Papanagnou D. Zooming-out COVID-19: Virtual clinical experiences in an emergency medicine clerkship. *Med Educ.* (2020) 54:1182–3. 10.1111/medu.14266 32502282 PMC7300610

[16] Meny L de Voest M Salvati L. Assessment of student pharmacist learning within an interprofessional simulation: A comparison of small group vs. large group debriefing. *Curr Pharm Teach Learn.* (2019) 11:533–7. 10.1016/j.cptl.2019.02.007 31171257

[17] Eppich W Hunt E Duval-Arnould J Siddall V Cheng A. Structuring feedback and debriefing to achieve mastery learning goals. *Acad Med.* (2015) 90:1501–8. 10.1097/ACM.0000000000000934 26375272

[18] Khan R Atta K Sajjad M Jawaid M. Twelve tips to enhance student engagement in synchronous online teaching and learning. *Med Teach.* (2022) 44:601–6. 10.1080/0142159X.2021.1912310 33877950

[19] Lee J Lee H Kim S Choi M Ko I Bae J Debriefing methods and learning outcomes in simulation nursing education: A systematic review and meta-analysis. *Nurse Educ Today.* (2020) 87:104345. 10.1016/j.nedt.2020.104345 32135455

[20] Apfelbaum J Hagberg C Connis R Abdelmalak B Agarkar M Dutton R 2022 American society of anesthesiologists practice guidelines for management of the difficult airway. *Anesthesiology.* (2022) 136:31–81. 10.1097/ALN.0000000000004002 34762729

[21] Cumin D Weller J Henderson K Merry A. Standards for simulation in anaesthesia: Creating confidence in the tools. *Br J Anaesth.* (2010) 105:45–51. 10.1093/bja/aeq095 20507857

[22] Mian A Khan S. Medical education during pandemics: A UK perspective. *BMC Med.* (2020) 18:100. 10.1186/s12916-020-01577-y 32268900 PMC7141929

[23] Murdock H Penner J Le S Nematollahi S. Virtual morning report during COVID-19: A novel model for case-based teaching conferences. *Med Educ.* (2020) 54:851–2. 10.1111/medu.14226 32403168 PMC7273056

[24] Su B Zhang T Yan L Huang C Cheng X Cai C Online medical teaching in china during the Covid-19 pandemic: Tools, modalities, and challenges. *Front Public Health.* (2021) 9:797694. 10.3389/fpubh.2021.797694 34988057 PMC8720748

[25] Sung T Hsu H. Improving critical care teamwork: Simulation-based interprofessional training for enhanced communication and safety. *J Multidiscip Healthc.* (2025) 18:355–67. 10.2147/JMDH.S500890 39872869 PMC11769723

[26] McDonald E Boulton J Davis JL. E-learning and nursing assessment skills and knowledge - An integrative review. *Nurse Educ Today.* (2018) 66:166–74. 10.1016/j.nedt.2018.03.011 29705504

[27] Rochlen L Housey M Gannon I Mitchell S Rooney D Tait A Assessing anesthesiology residents’ out-of-the-operating-room (OOOR) emergent airway management. *BMC Anesthesiol.* (2017) 17:96. 10.1186/s12871-017-0387-2 28709415 PMC5512836

[28] Nuampa S Ratinthorn A Tangsuksan P Chalermpichai T Kuesakul K Ruchob R Factors influencing critical thinking in simulation-based maternal-child nursing education among undergraduate nursing students: A mixed methods study. *BMC Nurs.* (2025) 24:389. 10.1186/s12912-025-03016-w 40197388 PMC11978189

[29] Klasen J Meienberg A Bogie B. Medical student engagement during COVID-19: Lessons learned and areas for improvement. *Med Educ.* (2021) 55:115–8. 10.1111/medu.14405 33141957

[30] Cass G Crofts J Draycott T. The use of simulation to teach clinical skills in obstetrics. *Semin Perinatol.* (2011) 35:68–73. 10.1053/j.semperi.2011.01.005 21440813

[31] Cant R Cooper S. Simulation in the Internet age: The place of web-based simulation in nursing education. An integrative review. *Nurse Educ Today.* (2014) 34:1435–42. 10.1016/j.nedt.2014.08.001 25156144

[32] Selcuk A Ozturk N Onal N Bozkir A Aksoy N. Online simulation versus traditional classroom learnings in clinical pharmacy education: Effect on students’ knowledge, satisfaction and self-confidence. *BMC Med Educ.* (2025) 25:437. 10.1186/s12909-025-07028-7 40133885 PMC11938679

[33] Webster D. Using standardized patients to teach therapeutic communication in psychiatric nursing. *Clin Simulat Nurs.* (2014) 10:e81–6. 10.1016/j.ecns.2013.08.005

[34] Bindoff I Ling T Bereznicki L Westbury J Chalmers L Peterson G A computer simulation of community pharmacy practice for educational use. *Am J Pharm Educ.* (2014) 78:168. 10.5688/ajpe789168 26056406 PMC4453084

[35] Adsız M Dinçer S. The analysis of classroom management challenges faced by teachers in online classrooms. *TechTrends.* (2025) 69:345–61. 10.1007/s11528-025-01042-8

[36] Woda A Johnson B Hansen J Chen K Dreifuerst K. The importance of feedback with an asynchronous online training program when learning debriefing for meaningful learning. *Clin Simulat Nurs.* (2025) 101:101709. 10.1016/j.ecns.2025.101709

[37] Decker S Sapp A Bibin L Brown M Crawford S Jabeen Fayyaz J The impact of the simulation debriefing process on learning outcomes – an umbrella review protocol. *Clin Simulat Nurs.* (2024) 89:101505. 10.1016/j.ecns.2023.101505

[38] Stokes-Parish J Duvivier R Jolly B. Investigating the impact of moulage on simulation engagement–A systematic review. *Nurse Educ Today.* (2018) 64:49–55. 10.1016/j.nedt.2018.01.003 29459192

