# Supplementary material for: Online scenario simulation teaching in airway management for undergraduate anesthesia students
Source: Front Med (Lausanne). 2025 Jun 10;12:1563540. doi: 10.3389/fmed.2025.1563540 (PMC12185409; doi:10.3389/fmed.2025.1563540)

大連醫科大學附屬第一醫院
THE FIRST AFFILIATED HOSPITAL OF DALIAN MEDICAL UNIVERSITY

Online scenario simulation teaching in airway management for undergraduate anesthesia students

服务、创意，做什么都要好
聚焦医疗前沿，注重患者感受
弘道笃行 精诚大医
建设国内一流、国际知名的 医疗研究型大学附属医院

In this presentation, we will provide a demonstration some of online scenario simulation teaching components discussed in the article.

Online scenario simulation teaching for difficult airway management during thyroid surgery.

Patient Cases

- A 60-year-old woman.
- Health ,no abnormalities in all examinations.
- Radical surgery for thyroid cancer under general anesthesia.

13:30 Upon arrival in the operating room, Bp 120/80 mmHg, HR 80 bpm, SpO_2 96%.

13:35 Oxygen was supplied via a mask at a flow rate of 6 /min, which successfully increased the SpO_2 to 98%. Anesthesia was induced three minutes later using a combination of midazolam 1 mg, propofol 100 mg, sufentanil 20 μg, and rocuronium bromide 45 mg.

13:42 A direct laryngoscopic intubation was performed.

13:43 Post-laryngoscopic Visualization of Vocal Folds Reveals the Following Image:

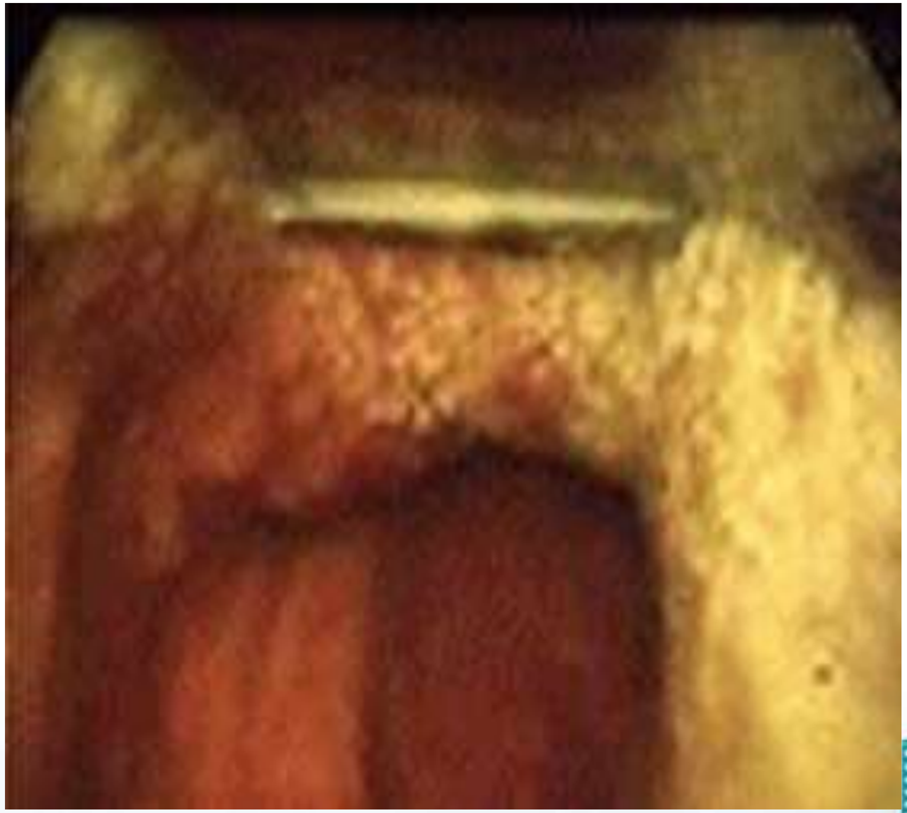

What are you gonna do?

Following the unsuccessful attempt at intubation with a standard laryngoscope, a video laryngoscope was employed in an effort to improve visualization. However, despite these efforts, the vocal folds could not be adequately exposed, resulting in a continued failure to intubate and a consequent decline in oxygen saturation levels.

13:50

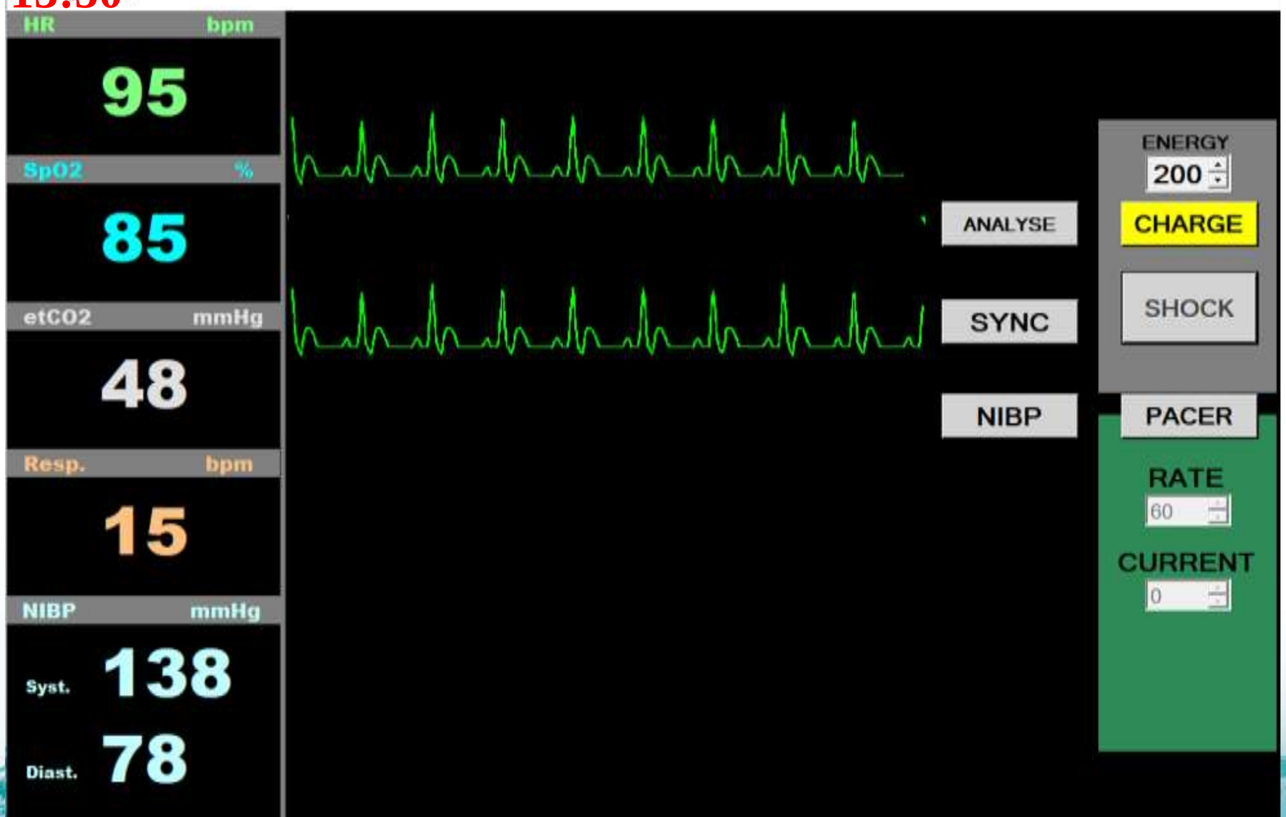

What are you gonna do?

After withholding intubation for mask ventilation, the vital signs were as follows:

13:55

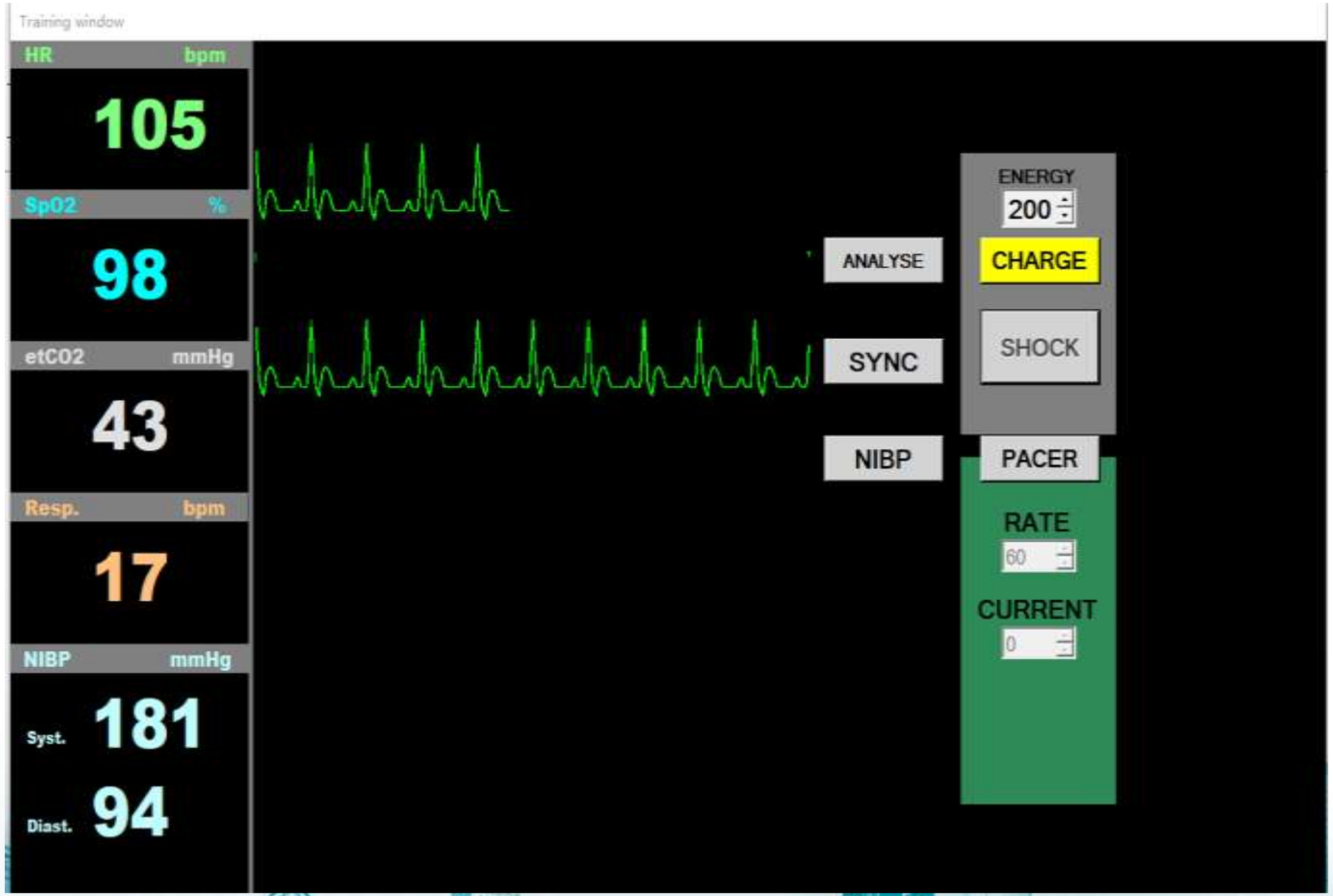

What are you gonna do?

14:00 Post-treatment, the patient's vital signs were stable.

14:06 A subsequent attempt to intubate using a video laryngoscope was unsuccessful. Consequently, a face mask was utilized for manual ventilation. This intervention was met with increased resistance to air entry, and auscultation revealed crackling sounds (rales) in both lungs. Additionally, there was a notable decline in oxygen saturation levels.

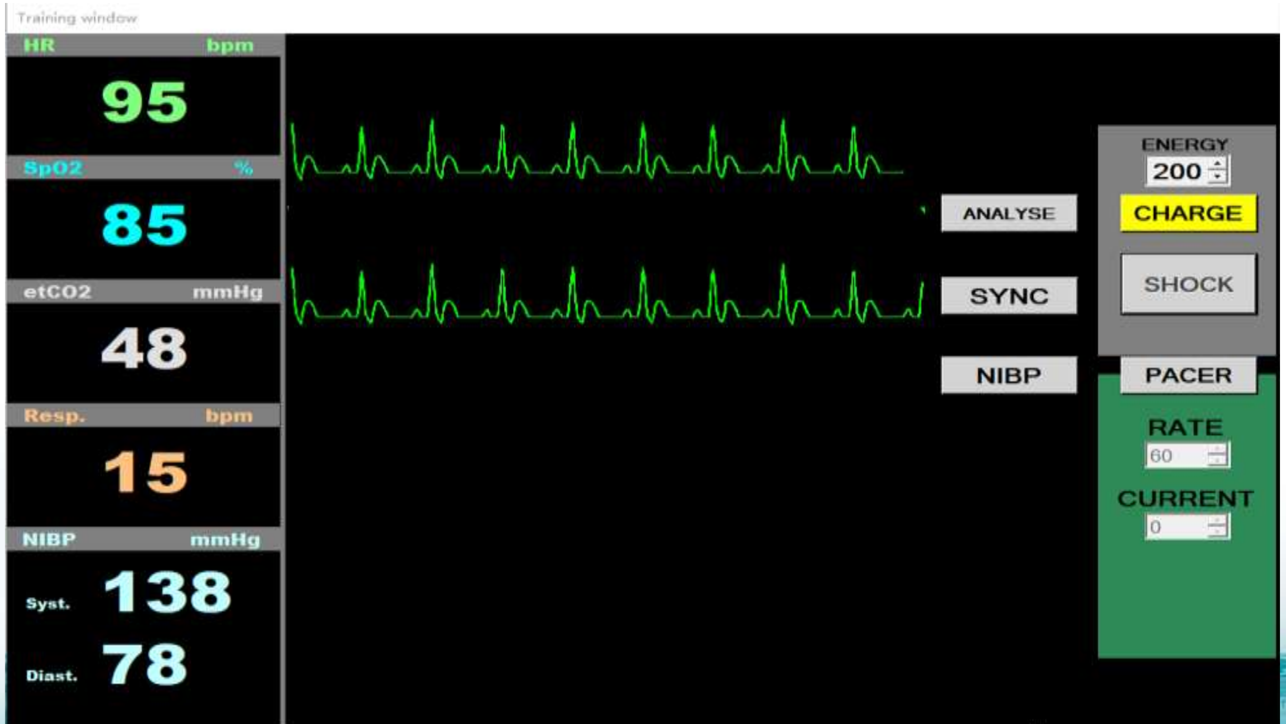

What happened to the patient?

What are you gonna do?

Call the supervising physician , and what medication would you use to relieve bronchospasm?

14:15

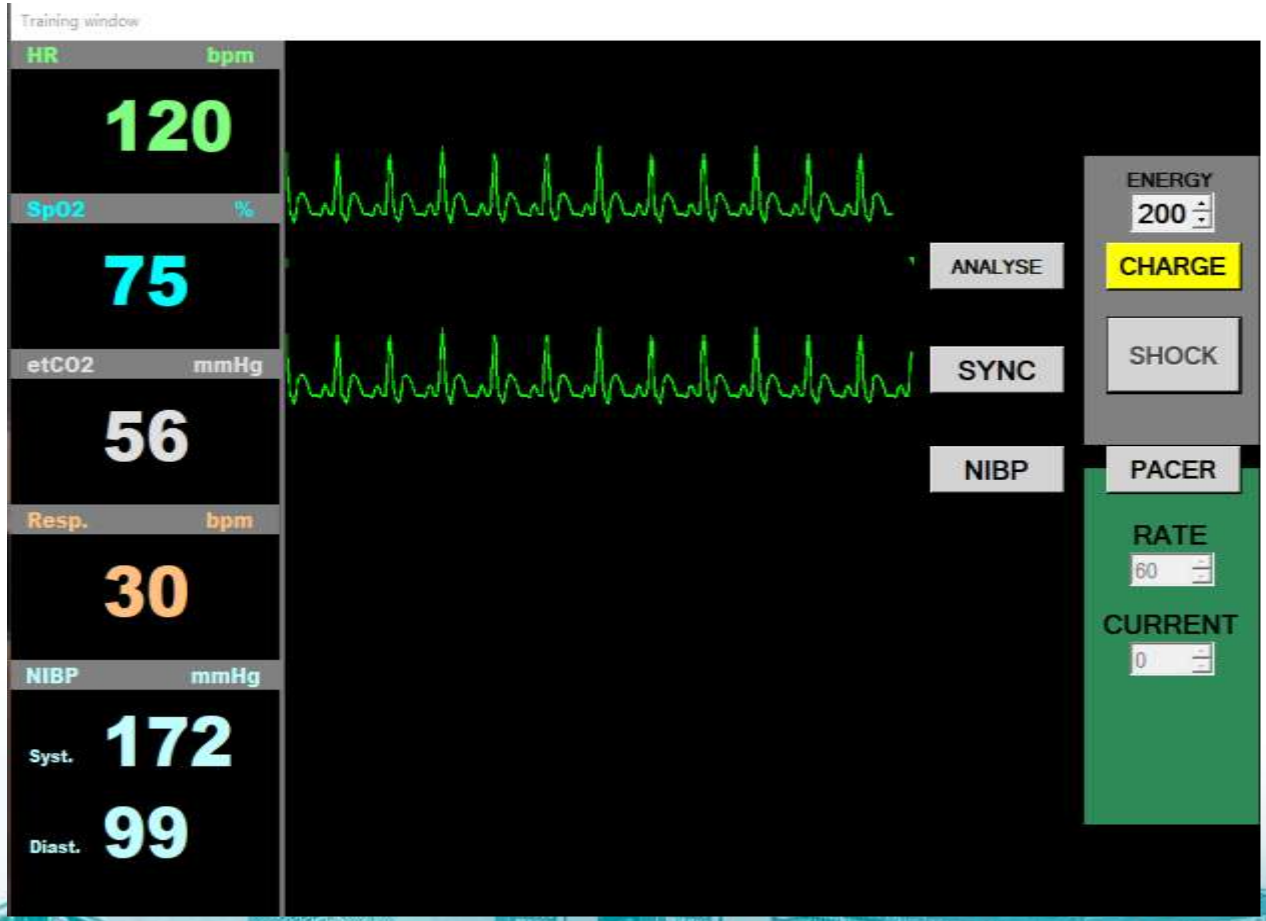

What are you gonna do?

14:25 Following all attempts, the patient's vital signs were successfully stabilized, and the supervising physician successfully performed the tracheal intubation.

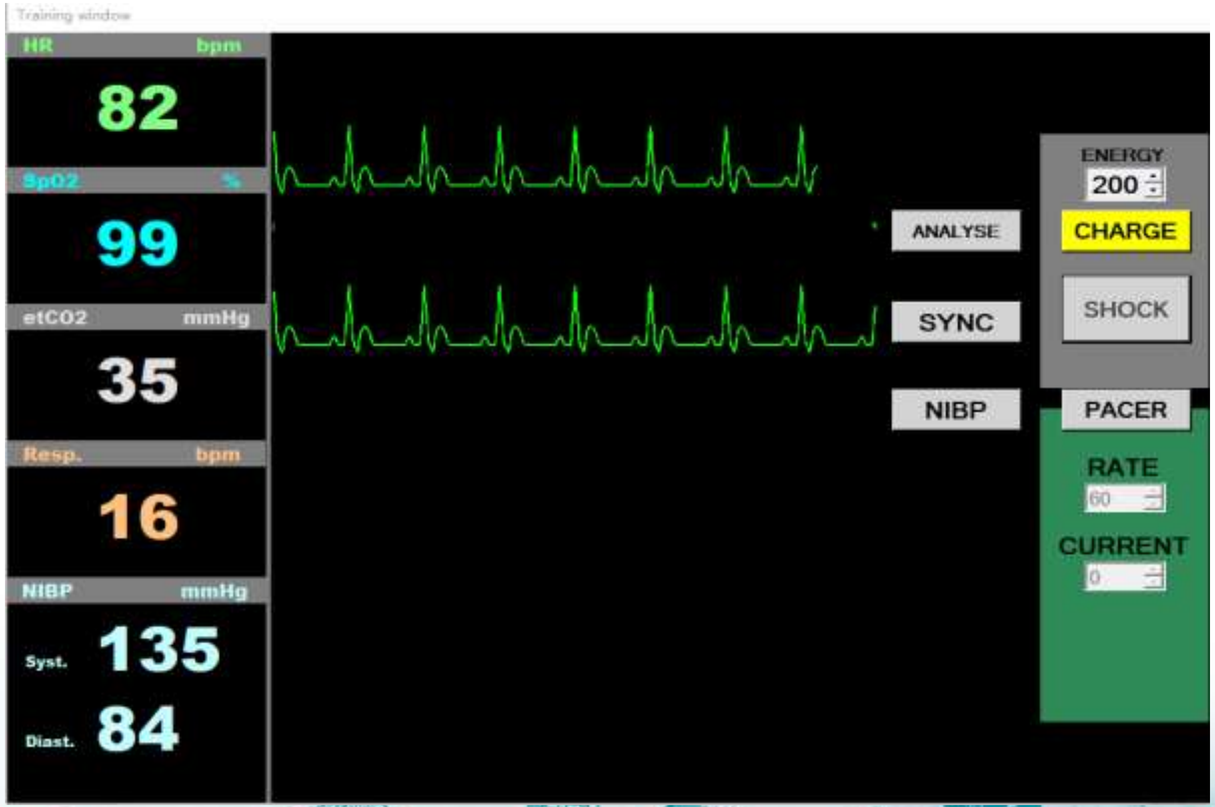

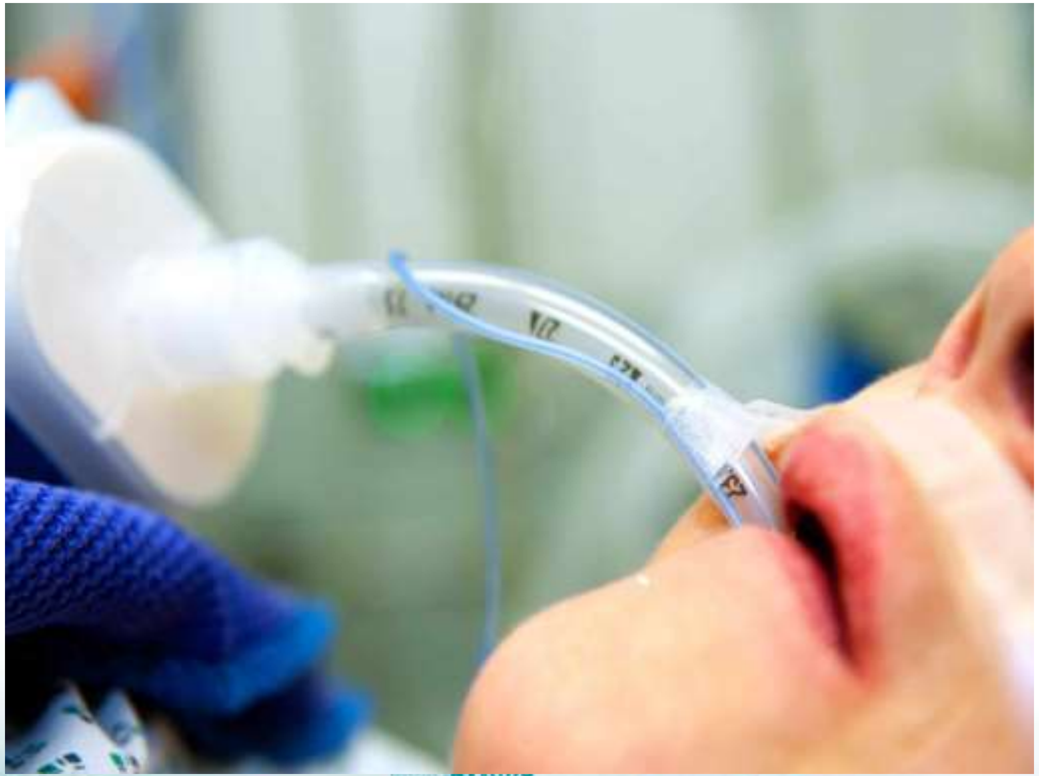

Supplement: Supplementary file 1 [file Data_Sheet_1.pdf]
