# Supplementary material for: Online scenario simulation teaching in airway management for undergraduate anesthesia students
Source: Front Med (Lausanne). 2025 Jun 10;12:1563540. doi: 10.3389/fmed.2025.1563540 (PMC12185409; doi:10.3389/fmed.2025.1563540)
Supplement: Supplementary file 2 [file Data_Sheet_2.pdf]

Supplementary Material 2. Standardized non-technical skills assessment

Assessment category	Assessment criteria	Score
Task Management	Tasks are allocated reasonably with clear priorities	
	Equipment is prepared and procedures are performed skillfully	
	Multiple tasks are managed effectively without confusion	
Teamwork	Good collaboration among team members with clear division of labor	
	Effective communication to avoid misunderstandings	
	Plays an active role in the team, supporting other members	
Communication	Information is conveyed clearly and accurately	
	Effectively listens to others' opinions	
	Effectively communicate critical information in emergencies	
Sustained Vigilance	Continuous attention to the patient's condition without negligence	
	Timely identification of potential issues	
	Maintains focus during prolonged operations	
Crisis Identification	Rapid identification of crisis situations	
	Timely measures taken to address crises	
	Remains calm in crisis situations	
Decision Making	Can make sound decisions quickly	
	Adjust decisions based on circumstances	
	Can explain the basis for decisions	
Self-Confidence	Confidence in own skills and decisions	
	Maintains confidence under pressure	
	Learn from mistakes and makes improvements	
